# Pigs, Biomes, and the Pork Peril: A One Health and One Welfare Meta-Narrative

**DOI:** 10.53941/dbgs.2026.100004

**Published:** 2026-07-24

**Authors:** Niyaz Ahmed

**Affiliations:** Pathogen Biology Laboratory, Department of Biotechnology and Bioinformatics, University of Hyderabad, Hyderabad 500046, India

**Keywords:** swine production, zoonoses, antimicrobial resistance, climate risk, cancer risk

## Abstract

Global pork production and consumption occupy a dominant yet insufficiently scrutinized position within modern food systems, with 1.5 billion pigs slaughtered each year, a staggering loss of animal life. This perspective synthesizes published evidence and opinion from microbiology, epidemiology, environmental science, and public health to examine risks associated with industrial pig production, including zoonotic pathogen emergence, antimicrobial resistance (AMR), environmental degradation, and chronic disease burdens. The classification of processed meat as carcinogenic by the World Health Organization reinforces the need for critical reassessment of pork-centric production and consumption systems. High-density pig farming amplifies microbial spillover, ecological pressure, and public health vulnerability through intensive resource use, antibiotic dependence, and waste externalities. The convergence of these risks within industrial swine systems warrants particular attention. Accordingly, transitioning both industrial pig production and processing toward less risk-intensive and more adaptable alternatives based on diversified, agroecological food systems entailing aquaculture, sustainable dairying, poultry, and plant-based food cultivation can provide pragmatic pathways to resilient, health-conscious, and sustainable food supplies that operate within planetary boundaries. This direction aligns with sustainable food policies and frameworks advanced by international governing bodies and directly operationalizes the integrated One Health and One Welfare frameworks.

## 1. The Overlooked Toll

In October 2015, the World Health Organization’s International Agency for Research on Cancer (WHO/IARC) published a monograph detailing the carcinogenic risks associated with red and processed meats [1]. Globally, pork accounts for approximately 34% of total meat consumption [2], while products derived from swine constitute up to 64% of the processed meat market share [3]. When we refer to processed meat, it most predominantly includes pork products (bacon, sausage, hot dogs, pepperoni, ham, and cold cuts like bologna and salami) [3].

The WHO/IARC monograph classified processed meat, of which pork products form the predominant component, as a group-1 carcinogen, placing it in the same category as tobacco in terms of established carcinogenicity [1]. A decade later, researchers in the United Kingdom have called for the introduction of cigarette-style health warnings on bacon and ham packaging, citing their potential association with increased bowel cancer risk [4]. Due to non-availability of such a labeling system warning consumers about pork’s inherent risks and nitrite use in processed pork, about 54,000 Britons over the years developed bowel cancer, which has cost the NHS about £3bn [4]. In California, the labeling of bacon is subject to the state’s Proposition 65 law, which requires warning labels for processed meats, such as bacon.

Pork remains one of the most widely consumed meats worldwide; however, its production and consumption present a range of significant biological and environmental challenges that have largely been ignored and warrant urgent attention.

Beyond its carcinogenic classification, the rapidly expanding swine production industry carries far-reaching implications, including contributions to pathogen emergence, antimicrobial resistance (AMR), adverse effects on gut health, elevated risks of cancer and other chronic diseases, environmental pollution, and substantial role in global carbon emissions.

## 2. Epidemiological Overview

Several pathogenic bacteria and parasites including *Trichinella spiralis* (which causes trichinosis), *Taenia solium* (the pork tapeworm responsible for taeniasis and neurocysticercosis), *Toxoplasma gondii*, *Leptospira* spp., *Listeria monocytogenes*, *Streptococcus suis, Salmonella* spp., *Campylobacter jejuni*, *Brucella* spp., *Clostridioides difficile, Clostridium botulinum*, and *Yersinia enterocolitica* may be associated with pork production and consumption, among other food-animal and environmental reservoirs [5–7]. Decades of epidemiological studies and outbreak reports consistently point towards pork as a significant source of human infection with these agents.

Additionally, pork is a source of broad-host-range enteric pathogens with wide host tropism such as *Salmonella*. Their presence in pork is a critical zoonotic risk whereby the infection is acquired mainly via fecal-oral routes involving pigs, with latent carriers shedding bacteria during stressful transport and slaughter conditions and that it contaminates multiple tissues, including lymph nodes and intestinal tract, escalating carcass contamination risks during processing [8–10]. The evolution and global spread of swine-adapted extensively drug-resistant *Salmonella* strains are generally linked to intensive farming and pork trade [11].

Antibiotic residues found in pork may promote the growth of resistant gut bacteria, further complicating human health outcomes. Consequently, the consumption of predominantly processed pork is associated with health risks beyond infection including obesity and cardiovascular disease [12] and possibly via mechanisms tied to gut microbiota alterations and inflammation [13]. Moreover, heavy use of antibiotics in pig production leads to residues widely dispersed through manure and wastewater that permeate soils and water sources. This not only degrades microbial ecosystem integrity but also threatens drinking water safety and facilitates the proliferation of fecal bacteria and associated antimicrobial resistance genes (ARGs), leading to a significant crisis [14–16]. In addition, the pork industry also plays a major role in the global antimicrobial consumption quagmire, exacerbating AMR with grave public health implications. Worldwide, about 99,500 tons of antimicrobials were used in livestock in 2020, with pigs consuming a significant share estimated at roughly 172 mg per kg of animal weight, produced largely due to routine use of antibiotics as growth promoters, as well as for disease prevention, and toward therapeutic regimens in intensive farming systems [17,18]. This widespread use selects for resistant bacteria in swine, which can transfer to humans through contaminated meat, direct contact, or environmental pathways. For instance, groundwater and manure runoff spread these bacteria and ARGs further [14–16]. The global magnitude of AMR is devastating: bacterial resistance directly caused 1.27 million deaths in 2019, about 1.14 million in 2021 with an estimated 1.91 million deaths in 2050; projections warn of over 39 million direct deaths from 2025 to 2050 [19,20].

Pork-associated viruses significantly impact human health by acting as zoonotic disease agents capable of crossing species barriers and causing both acute and chronic illnesses. The list of swine origin viruses which could be associated with disease including potentially zoonotic infections or experimental infections is rather long and alarming: swine enteric alphacoronavirus (SeACoV), foot-and-mouth disease virus (FMDV), classical swine fever virus (CSFV), porcine epidemic diarrhea virus (PEDV), porcine reproductive and respiratory syndrome virus (PRRSV), porcine circovirus type 2 (PCV2), swine hepatitis E virus (swine HEV), swine influenza virus (SIV), porcine sapovirus (Porcine SaV), torque teno sus virus (TTSuV), African swine fever virus (ASFV), and Senecavirus A (SVA) [21]. Some of these viruses, even if not directly associated with human infections, could very well form the trajectory of future emerging viral diseases. Because swine serve as highly suitable permissive hosts [22], viral gene re-assortment within these animals can generate novel strains with pandemic potentials. Ultimately, the role of pigs as intermediates or amplifiers in emerging zoonotic virus spillovers is widely recognized in the literature and several of these viruses simultaneously carry devastating economic and veterinary public health impacts.

Pigs serve as great reservoirs of influenza viruses with extended host tropism and thereby infection risks in different species including humans, and pose pandemic risks [23]. From a biosecurity perspective, swine hosts represent a genuine threat posing as ‘mixing vessels’ capable of re-assorting avian, human, and swine influenza strains, promoting viral mutation and augmenting pandemic risks [23]. Other viruses such as Nipah cause deadly encephalitis. Though some swine-specific viruses, such as African swine fever, do not infect humans, they indirectly threaten food security and global economies. The most immediate and well-documented viral infection risk emanates from Hepatitis-E virus infection leading to hepatitis. Although it might be often asymptomatic or mild, it can lead to acute liver failure, especially in vulnerable populations [24].

The impact of pork on gut health goes beyond infections, as it also affects the very balance of the gut microbiome. Regular consumption, especially of processed pork products, has been linked to reduced microbial diversity with deleterious impact on inflammatory and metabolic pathways. The incidences of Type-2 diabetes and consumption of red meat, pork in particular, have risen simultaneously and consumption of processed meats have shown increased association with diabetes risk in observational studies [25]. Literature is replete with reports of elevated levels of trimethylamine-N-oxide (TMAO), fasting insulin, and C-reactive protein in association with pork consumption and pointing at increased inflammation, metabolic dysfunction, and an elevated risk for adverse cardiovascular events. High TMAO levels were observed in experimental animals fed by diets rich in pork meat and processed pork, together with western background diets [26]. A recent analysis of the data from large genome-wide association studies found that higher pork intake was significantly associated with increased risk of atrial fibrillation [27].

In the long term, and often in conjunction with other lifestyle factors, high intake of pork or its processed products can contribute to the development of Non-Alcoholic Fatty Liver Disease (NAFLD) and potentially other forms of chronic liver disease [28]. Some studies suggest a correlation between dietary pork and mortality due to cirrhosis. While the link is complex, there could be certain components in pork that likely exacerbate liver damage [29].

At the chemical level, components abundant in pork such as polycyclic aromatic hydrocarbons, heme iron, heterocyclic amines, and N-nitroso compounds have the potential to inflict oxidative damage on colon cells, contributing to the development of colorectal cancer [30–32]. Supporting these findings, animal studies reveal that pork proteins could drive oxidative stress and low-grade inflammation in the colon, disrupt beneficial bacteria, and alter gene expression related to immunity and metabolism [33]. Wary of these evidences, major health authorities classify processed meat as carcinogenic and red meat including pork as probably carcinogenic to humans [1]. Scientific studies have shown that eating large quantities of processed red meat, which predominantly includes pork, increases the risk of colorectal cancer. The evidence for processed pork products such as bacon and ham is stronger than for unprocessed cuts, but excessive consumption of either is associated with an increased risk of colorectal cancer and associated mortality [1,34].

Although the interaction of dietary proteins and autoimmunity is largely understudied, dietary proteins have been proposed as one of the risk factors for the autoimmune disorders. It has been reported that pig proteins indicated high number of unique autoimmune epitopes corresponding to human proteins [35]. A study reported that the abattoir workers while processing brains of pigs developed autoimmune neurological disorders [36] with IgG specific antibodies to myelin basic protein (MBP) found in 75% of workers [37]. It has been shown that numerous pig-derived autoimmune epitopes were specific to multiple sclerosis epitopes that were found to be expressed in all tissues of the human body and could be present similarly in pigs. Interestingly, pigs shared unique epitopes with antigens involved in other autoimmune disorders, such as Goodpasture syndrome, autoimmune atherosclerosis, dermatomyositis and bullous pemphigoid [35].

The environmental footprint and impact of global food production including pork production is substantial and mitigation through various measures is suggested [38]. Globally, producing 1 kg of pork meat emits up to 7 kg of CO_2_-equivalent as upper limit, with some systems reporting even higher emissions [39,40]. The vast majority of these emissions come from feed production and manure management, while processing and transportation contribute smaller, yet meaningful shares [39,40]. Beyond climate impacts, pig farming requires substantial land and water resources. It also contributes heavily to nutrient runoff causing eutrophication, soil and water contamination, and significant biodiversity loss [39]. The scale of production is staggering as between 1.3 and 1.5 billion pigs are slaughtered globally each year amplifying the environmental burdens tremendously [41,42]. To put this into perspective, pork’s footprint rivals that of chicken but is considerably far larger than plant-based protein sources like lentils in terms of greenhouse gas emissions, water use, and land occupation [38,39].

While organic pork production is often viewed as a more environmentally friendly choice, it still contributes significantly to greenhouse gas emissions due to feed intake and manure methane emissions coupled with lower yields [40,43]. Another critically underappreciated environmental hazard lies in the processing industry’s waste streams: offal, hides, discards, and manure. These byproducts frequently contaminate waterways, aquifers, and surrounding soils with excessive nutrients, harmful pathogens including antibiotic-resistant bacteria and toxic compounds [44]. Runoff from pig farms introduces high levels of nitrogen, phosphorus, heavy metals, and pathogens into local ecosystems, exacerbating eutrophication, oxygen depletion in aquatic systems, and jeopardizing crop and human health [14,15,44].

While it is worthwhile to mention the advances made in animal husbandry, biosecurity, and regulatory oversight, as well as pork’s nutritional value as a source of protein and fat, and asserted production efficiencies, including potential land-use reductions and farming-related benefits to rural livelihoods, these advantages are insufficient to offset the well-documented health and environmental risks associated with modern industrial pork production. It is possible that some phrases and idioms will obscure environmental and health impacts of intensive pork production. Notions such as ‘feed the world’ and ‘regenerative agriculture’ frame industrial pork as necessary or sustainable, while claims about production efficiency per unit of meat distract from the massive overall emissions, waste, and resource use. Manure is sometimes lauded as ‘black gold’, yet in reality, large-scale waste management poses serious pollution risks. By downplaying absolute emissions and methane contributions, these narratives shield intensive swine farming from meaningful climate accountability.

From a cultural and psychological perspective, pork consumption elicits strong aversions within many religiously observant and aesthetically sensitive communities. The famous ‘yuck factor’, associated with pigs’ omnivorous behavior (and coprophagy emanating from their long-standing associations with filth) remains influential, with approximately 30–40% of the global population abstaining from pork for religious, cultural, or personal reasons. Major world religions and cultural traditions explicitly prohibit pork consumption [45,46].

## 3. The Path Forward

The converging infection risks and zoonotic hazards, biosecurity imperatives, the massive ecological footprint, as well as significant cultural avoidance collectively paint pork, whether conventionally or organically produced, as a problematic and high-risk food commodity [5,6,9,10,13,45,46]. The accumulating evidence from microbiology, epidemiology, environmental science, and social research underscores the necessity for global societies to consider transitioning away from pork consumption. Simply mitigating impacts through incremental measures falls short of resolving the multifaceted challenges or successfully operationalizing the tenets of the One Health and One Welfare frameworks.

Shifting to alternate protein sources aligns powerfully with international sustainability goals. The most evidence-based, health-promoting, and ecologically sustainable path forward lies in transition toward healthier and more affordable alternatives such as seafood, poultry, dairy, and vegetables. The available evidence supports reducing dependence on intensive pork production systems and encouraging diversified dietary and agricultural transitions that may mitigate environmental and public health burdens. It supports the United Nations’ Sustainable Development Goals such as (a) responsible consumption & production (SDG 12), (b) climate action (SDG 13), (c) life below water (SDG 14), and (d) life on land (SDG 15) by reducing resource-intensive livestock pressures and mitigating biodiversity loss. Such a shift would align with and reinforce the European Union’s *Green Deal* and *Farm to Fork* strategies, which prioritize nutritious, sustainable diets and aim to curb AMR and agricultural environmental impacts [46–48]. It is estimated that replacing animal-based foods including pork with safe alternatives could sharply cut food’s environmental impact in terms of global agricultural land use, water demand, greenhouse gas emissions, and biodiversity decline and would pave the Paris Agreement’s 1.5 °C pathway further on [38,49].

There could be some reservations to the opinions presented herein. For instance, infestations, such as trichinellosis, might be reduced simply through proper cooking. In addition, any intensive farming system—whether pigs, poultry, or cattle—brings its own environmental and zoonotic challenges. So, while it is important to recognize that all livestock systems carry some level of zoonotic risks, pigs stand out scientifically and epidemiologically. Due to their unique biology, microbial spillovers, environmental stresses, and public-health vulnerabilities integrate in particularly potent ways. This integrative convergence is intensified by their physiological resemblance to humans and their ability to harbor and amplify a wide range of pathogens. Furthermore, the fact that pork surpasses the most heavily industrially processed meat classes introduces additional nutritional and chemical concerns that extend beyond the farming stage [1,7,43,46].

Finally, lumping all red meats together discourages a more nuanced conversation about the individual and critical impacts such as those of pork. The ruminant and poultry production systems form backbones of some of the major agrarian economic systems; they are biologically safer, aesthetically and culturally more acceptable and yield valuable co-products such as milk, hides, fibers, or eggs. Pork production, on the other hand, is almost exclusively meat-focused, rendering it economically narrow and environmentally costly. Phasing down industrial pork production and aligning transitions with the Food and Agriculture Organization’s Strategic Framework (2022–2031), the One Health Initiative approach, and recommendations from IPES-Food might facilitate a shift away from narrowly optimized, high-intensity production models toward more circular, diversified, and context-sensitive systems. In this context, greater emphasis on well-managed aquaculture, sustainable poultry, diversified dairying systems, and vegetable-based food systems offers viable pathways for reducing environmental pressures while maintaining nutritional security. This shift supports international policies on sustainable livestock transformation, which advocate for reducing the ecological footprint of high-density farming to mitigate zoonotic disease risks and AMR. Consequently, phasing out industrial pig farming and processing in favour of the above-mentioned alternative food production systems in a conscious and prudent manner represents a practical and policy-viable path forward. This will allow us to advance in the context of the IPES-Food and UN goals of building resilient, diversified supply chains that stay within planetary boundaries while ensuring long-term global food security.

## Data Availability

There were no sharable data generated as a part of this publication since it is an opinionated article.

## References

[1] Bouvard V; Loomis D; Guyton KZ; Carcinogenicity of consumption of red and processed meat. Lancet Oncol. 2015, 16, 1599–1600. 26514947 10.1016/S1470-2045(15)00444-1

[2] Kim SW; Gormley A; Jang KB; Invited Review: Current status of global pig production: An overview and research trends. Anim Biosci. 2024, 37, 719–729. 37946421 10.5713/ab.23.0367 PMC11016693

[3] Processed Meat Market Size & Share Analysis—Growth Trends and Forecast (2025–2030). Available online: https://www.mordorintelligence.com/industry-reports/global-processed-meat-market-industry (accessed on 1 July 2026).

[4] The Guardian. Scientists Demand Cancer Warnings for Bacon and Ham in the UK. Available online: https://www.theguardian.com/society/2025/oct/24/scientists-demand-cancer-warnings-bacon-ham-uk (accessed on 1 July 2026).

[5] Lin C-N; Okabayashi T; Tummaruk P; Editorial: Zoonotic diseases among pigs. Front. Vet. Sci. 2023, 9, 1122679. 10.3389/fvets.2022.1122679. 36686156 PMC9851072

[6] Augustyniak A; Pomorska-Mól M. An Update in Knowledge of Pigs as the Source of Zoonotic Pathogens. Animals 2023, 13, 3281. 10.3390/ani13203281. 37894005 PMC10603695

[7] Baer AA; Miller MJ; Dilger AC. Pathogens of Interest to the Pork Industry: A Review of Research on Interventions to Assure Food Safety. Compr. Rev. Food Sci. Food Saf. 2013, 12, 183–217. 10.1111/1541-4337.12001.

[8] Abrego A; Wiseman B; Gragg SE. Summarizing the Current knowledge and Existing Knowledge Gaps for Preharvest and Postharvest Salmonella Contamination in Pork. J. Food Prot. 2025, 88, 100609. 40876777 10.1016/j.jfp.2025.100609

[9] Martins BTF; Camargo AC; Tavares RM; Relevant foodborne bacteria associated to pork production chain. Adv. Food Nutr. Res. 2025, 113, 181–218. 40023561 10.1016/bs.afnr.2024.09.016

[10] Jaramillo-Bedoya E; Flórez-Elvira LJ; Ocampo-Ibáñez ID. High Prevalence of Salmonella spp. in Ready-to-Eat Artisanal Pork Sausages Sold at Food Outlets in Quindío, Colombia. Pathogens 2025, 14, 31. 39860992 10.3390/pathogens14010031 PMC11768352

[11] Prasertsee T; Prachantasena S; Tantitaveewattana P; Assessing antimicrobial resistance profiles of Salmonella enterica in the pork production system. J. Med. Microbiol. 2024, 73, 001894. 39320348 10.1099/jmm.0.001894 PMC11423857

[12] Kennedy J; Alexander P; Taillie LS; Estimated effects of reductions in processed meat consumption and unprocessed red meat consumption on occurrences of type 2 diabetes, cardiovascular disease, colorectal cancer, and mortality in the USA: A microsimulation study. Lancet Planet Health 2024, 8, e441–e451. 38969472 10.1016/S2542-5196(24)00118-9

[13] Monger XC; Gilbert AA; Saucier L; Antibiotic Resistance: From Pig to Meat. Antibiotics 2021, 10, 1209. 34680790 10.3390/antibiotics10101209 PMC8532907

[14] Pham-Duc P; Nguyen-Viet H; Luu-Quoc T; Understanding antibiotic residues and pathogens flow in livestock wastewater from Smallholder Pig Farms to Agriculture Field in Ha Nam Province, Vietnam. Environ. Health Insights 2020, 14, 117. 10.1177/1178630220943206 PMC7543113 33088179

[15] Rasschaert G; Elst DV; Colson L; Antibiotic Residues and Antibiotic-Resistant Bacteria in Pig Slurry used to Fertilize Agricultural Fields. Antibiotics 2020, 9, 34. 31963594 10.3390/antibiotics9010034 PMC7168310

[16] Krapac IG; Dey WS; Roy WR; Impacts of swine manure pits on groundwater quality. Environ. Pollut. 2002, 120, 475–492. 12395861 10.1016/s0269-7491(02)00115-x

[17] Tiseo K; Huber L; Gilbert M; Global Trends in Antimicrobial Use in Food Animals from 2017 to 2030. Antibiotics 2020, 9, 918. 33348801 10.3390/antibiotics9120918 PMC7766021

[18] Van Boeckel TP; Brower C; Gilbert M; Global trends in antimicrobial use in food animals. Proc. Natl. Acad. Sci. USA 2015, 112, 5649–5654. 25792457 10.1073/pnas.1503141112 PMC4426470

[19] Naghavi M; Vollset SE; Ikuta KS; Global Burden of Bacterial Antimicrobial Resistance 1990–2021: A systematic analysis with forecasts to 2050. Lancet 2024, 404, 1199–1226. 39299261 10.1016/S0140-6736(24)01867-1 PMC11718157

[20] Murray CJ; Ikuta KS; Sharara F; Global burden of bacterial antimicrobial resistance in 2019: A systematic analysis. Lancet 2022, 399, 629–655. 35065702 10.1016/S0140-6736(21)02724-0 PMC8841637

[21] Meng XJ; Thiel V. Emerging and re-emerging porcine viruses. Virus Res. 2020, 290, 198198. 33065125 10.1016/j.virusres.2020.198198 PMC7553064

[22] Ma W; Kahn RE; Richt JA. The pig as a mixing vessel for influenza viruses: Human and veterinary implications. J. Mol. Genet. Med. 2008, 3, 158–166. 19565018 PMC2702078

[23] Ma W; Loving CL; Driver JP. From Snoot to Tail: A Brief Review of Influenza Virus Infection and Immunity in Pigs. J. Immunol. 2023, 211, 1187–1194. 37782856 10.4049/jimmunol.2300385 PMC10824604

[24] Sooryanarain H; Meng XJ. Swine hepatitis E virus: Cross-species infection, pork safety and chronic infection. Virus Res. 2020, 284, 197985. 32333941 10.1016/j.virusres.2020.197985 PMC7249539

[25] Stettler N; Murphy MM; Barraj LM; Systematic review of clinical studies related to pork intake and metabolic syndrome or its components. Diabetes Metab. Syndr. Obes. Targets Ther. 2013, 6, 347–357. 10.2147/DMSO.S51440 PMC3792009 24106428

[26] Thøgersen R; Rasmussen MK; Sundekilde UK; Background Diet Influences TMAO Concentrations Associated with Red Meat Intake without Influencing Apparent Hepatic TMAO-Related Activity in a Porcine Model. Metabolites 2020, 10, 57. 32041174 10.3390/metabo10020057 PMC7074160

[27] Li H; Xu J. Multifaceted dietary exposures and atrial fibrillation: Bidirectional causal evidence from two-sample Mendelian randomization study. Medicine 2025, 104, e44722. 40988167 10.1097/MD.0000000000044722 PMC12459541

[28] Ivancovsky-Wajcman D; Fliss-Isakov N; Grinshpan LS; High Meat Consumption Is Prospectively Associated with the Risk of Non-Alcoholic Fatty Liver Disease and Presumed Significant Fibrosis. Nutrients 2022, 14, 3533. 36079791 10.3390/nu14173533 PMC9459934

[29] Nanji AA; French SW. Relationship between pork consumption and cirrhosis. Lancet 1985, 325, 681–683. 10.1016/s0140-6736(85)91338-8 2858627

[30] Bastide NM; Pierre FH; Corpet DE. Heme iron from meat and risk of colorectal cancer: A meta-analysis and a review of the mechanisms involved. Cancer Prev. Res. 2011, 4, 177–184. 10.1158/1940-6207.CAPR-10-0113 21209396

[31] Turner ND; Lloyd SK. Association between red meat consumption and colon cancer: A systematic review of experimental results. Exp. Biol. Med. 2017, 242, 813–839. 10.1177/1535370217693117 PMC5407540 28205448

[32] Wang X; Lin X; Ouyang YY; Red and processed meat consumption and mortality: Dose-response meta-analysis of prospective cohort studies. Public Health Nutr. 2016, 19, 893–905. 26143683 10.1017/S1368980015002062 PMC10270853

[33] Zhang M; Zou X; Zhao D; Pork Meat Proteins Alter Gut Microbiota and Lipid Metabolism Genes in the Colon of Adaptive Immune-Deficient Mice. Mol. Nutr. Food Res. 2020, 64, e1901105. 32249499 10.1002/mnfr.201901105

[34] Sinha R; Cross AJ; Graubard BI; Meat intake and mortality: A prospective study of over half a million people. Arch. Intern. Med. 2009, 169, 562–571. 19307518 10.1001/archinternmed.2009.6 PMC2803089

[35] Gershteyn IM; Ferreira LMR. Immunodietica: A data-driven approach to investigate interactions between diet and autoimmune disorders. J. Transl. Autoimmun. 2019, 1, 100003. 32743493 10.1016/j.jtauto.2019.100003 PMC7388395

[36] Holzbauer SM; DeVries AS; Sejvar JJ; Epidemiologic investigation of immune-mediated polyradiculoneuropathy among abattoir workers exposed to porcine brain. PLoS ONE 2010, 5, e9782. 20333310 10.1371/journal.pone.0009782 PMC2841649

[37] Lachance DH; Lennon VA; Pittock SJ; An outbreak of neurological autoimmunity with polyradiculoneuropathy in workers exposed to aerosolized porcine neural tissue: A descriptive study. Lancet Neurol. 2010, 9, 55–66. 19945916 10.1016/S1474-4422(09)70296-0

[38] Poore J; Nemecek T. Reducing food’s environmental impacts through producers and consumers. Science 2018, 360, 987–992. 29853680 10.1126/science.aaq0216

[39] MacLeod M; Gerber P; Mottet A; Greenhouse Gas Emissions from Pig and Chicken Supply Chains: A Global Life Cycle Assessment; Food and Agriculture Organization: Rome, Italy, 2013.

[40] Yang P; Yu M; Ma X; Carbon Footprint of the Pork Product Chain and Recent Advancements in Mitigation Strategies. Foods 2023, 12, 4203. 38231615 10.3390/foods12234203 PMC10706032

[41] Faunalytics. Global Pig Slaughter Statistics and Charts. Available online: https://faunalytics.org/global-pig-slaughter-statistics-and-charts/ (accessed on 1 July 2026).

[42] OECD/FAO. OECD-FAO Agricultural Outlook 2025–2034; OECD/FAO: Paris, France; Rome, Italy, 2025.

[43] Kool A; Blonk H; Ponsioen T; Carbon Footprints of Conventional and Organic Pork: Assessments of Typical Production Systems in the Netherlands, Denmark, England and Germany; Blonk Milieu Advies: Gouda, Netherlands, 2009; p. 90.

[44] Zhang D; Wang X; Zhou Z. Impacts of Small-Scale Industrialized Swine Farming on Local Soil, Water and Crop Qualities in a Hilly Red Soil Region of Subtropical China. Int. J. Environ. Res. Public Health 2017, 14, 1524. 29211053 10.3390/ijerph14121524 PMC5750942

[45] Essig M. Lesser Beasts: A Snout-to-Tail History of the Humble Pig; Basic Books: New York, NY, USA, 2015.

[46] Ahmed N. The Last Pig—Ending the Pork Peril; Notion Press: Chennai, India, 2025.

[47] European Commission. Farm to Fork Strategy. Available online: https://food.ec.europa.eu/system/files/2020-05/f2f_action-plan_2020_strategy-info_en.pdf (accessed on 1 July 2026).

[48] Slow Food. What Do the New EU Farm to Fork and Biodiversity Strategies Mean for Slow Food? Available online: https://www.slowfood.com/wp-content/uploads/2023/12/F2F_Bio_Strat_Report_ENG.pdf (accessed on 1 July 2026).

[49] Humpenöder F; Popp A; Merfort L; Food matters: Dietary shifts increase the feasibility of 1.5 °C pathways in line with the Paris Agreement. Sci. Adv. 2024, 10, eadj3832. 38536907 10.1126/sciadv.adj3832 PMC10971398

